# A health equity research agenda for India: results of a consultative exercise

**DOI:** 10.1186/s12961-018-0367-0

**Published:** 2018-10-09

**Authors:** T.K. Sundari Ravindran, Tanya Seshadri

**Affiliations:** 1 Achutha Menon Centre for Health Science Studies, Sree Chitra Tirunal Institute for Medical Sciences & Technology, Trivandrum, Kerala India; 2 Independent researcher, Bengaluru, India

**Keywords:** Health equity research, AMCCON 2018, Priority setting exercise, Health inequities in India

## Abstract

**Background:**

This paper describes the process and outcome of a consultative exercise undertaken to develop a medium-term agenda for the next decade, and to identify a short list of immediate priorities for health equity research in India. This exercise was undertaken over 2014–2017 as part of ‘Closing the Gap: Health Equity Research Initiative in India’, implemented by the Achutha Menon Centre for Health Science Studies, at the Sree Chitra Tirunal Institute of Medical Sciences and Technology, Trivandrum, in south India.

**Methods:**

We adopted a five-step process for the agenda- and priority-setting exercise. The first step, which lasted for approximately 1 year, consisted of a synthesis of evidence on health inequities in India produced during 2000–2014 and identification of gaps. In the second step, we shared the evidence gaps identified and engaged with diverse stakeholders to develop the research agenda through face-to-face and online consultations. In step three, we consolidated the research agenda and identified continuing gaps. Key informant consultations by phone or email with experts in the areas where gaps were identified constituted the fourth step. In the fifth and final step, we organised an expert group consultation to review the agenda and identify immediate research priorities through a consensus process. Overall, approximately 220 persons participated in the entire process, and consisted of persons from diverse disciplines and sectors.

**Results:**

The research agenda and immediate priorities that emerged may be categorised into four themes, namely (1) descriptive research on the extent, nature and time trends in health inequities; (2) explanatory research on the pathways through which health inequities are created, and the political or policy environment that facilitates the process; (3) explanatory research that examines how health systems facilitate or mitigate inequities in healthcare; and (4) intervention research on initiatives that helped to mitigate health inequities, and examines the contributing factors.

**Conclusion:**

The strength of this research agenda is that it was developed through a broad-based consultation with stakeholders representing diverse disciplines, sectors and constituencies. The use of this agenda will help generate evidence that will facilitate India moving closer to the Sustainable Development Goal of leaving no one behind.

**Electronic supplementary material:**

The online version of this article (10.1186/s12961-018-0367-0) contains supplementary material, which is available to authorized users.

## Background

Equality in general and health equity in particular are key themes in the Sustainable Development Goals (SDG) agenda for 2030 [1]. Equality in the SDGs refers to the right to not be discriminated against. In the context of health, health equity is the desired goal because inequalities in health may arise from genetic, biological or random factors. Health equity is defined as “*unjust differences in health between persons of different social groups...* [which] *can be linked to disadvantages such as poverty, discrimination or lack of access to goods*” [2]. India’s National Health Policy 2017 also identified equity as a guiding principle [3]. Launching the new health policy in 2017, the Prime Minister of India stated: “*the National Health Policy marks a historic moment in our endeavour to create a healthy India where everyone has access to quality healthcare”* [4]. Programming for health equity calls for robust evidence on the concept, extent and nature of inequities in health, especially in view of scarce resources [5]. However, it is more important to generate evidence that unravels the factors and mechanisms that create, sustain and reinforce inequities. Nevertheless, the current evidence base on health inequities in India does not measure up to this task [6].

Disciplinary and sectoral boundaries fragment health equity research in India. Much of the academic research has been carried out by economists and public health researchers from a biomedical background, and each group has tended to examine issues from within its disciplinary perspective. A diverse group of civil society actors has also engaged in health equity research mainly to inform their field-based interventions and advocacy. There has been limited interaction between the academia and civil society actors working on health equity, with the subsequent absence of a community of health equity researchers to develop a common agenda [6]. One consequence of such fragmentation is the large research gap on important issues related to inequities in health in the country. Further, until recently, there have been no efforts to synthesise what is known and to develop a research agenda and priority areas of research on health inequities to inform national policy and programmatic action in India [6, 7].

In 2014, the Achutha Menon Centre for Health Science Studies at the Sree Chitra Tirunal Institute of Medical Sciences and Technology in south India, embarked on a project titled ‘Closing the Gap: Health Equity Research Initiative in India’. The overall aim of the initiative was to “contribute to the advancement of a sound and actionable evidence-base on inequities in health in India with a view to influence government and civil society initiatives to prioritise the reduction of health inequities [8].” This 4-year project is supported by the International Development Research Centre, Canada.

A critical task in the project was to develop a medium-term agenda for the next decade and identify a short list of immediate priorities for health equity research in India. The research agenda is meant primarily for use by researchers from diverse disciplines and sectors, interested in or working on health equity research. At the same time, the agenda aims to inform research funding and research policy-making. This paper describes the exercise undertaken and its outcomes.

## Methods

The methods adopted were informed by the following guiding principles, drawing on many years of experience in participatory research and action of key members of the Closing the Gap project, of which this activity was part.

- The agenda should focus on research that generates actionable evidence able to inform programming and policy-making to reduce health inequities, including increasing the visibility of health conditions and population groups about which/whom little is known
- The agenda-setting process should be consultative and iterative
- Multiple stakeholders should be involved in developing the agenda, including researchers as well as practitioners of diverse types such as policy-makers, programme managers, advocates and activists
- A conscious attempt should be made to represent the agendas of diverse marginalised groups
- The process should build on the already existing evidence base and draw on experiential knowledge

**Fig. 1:**
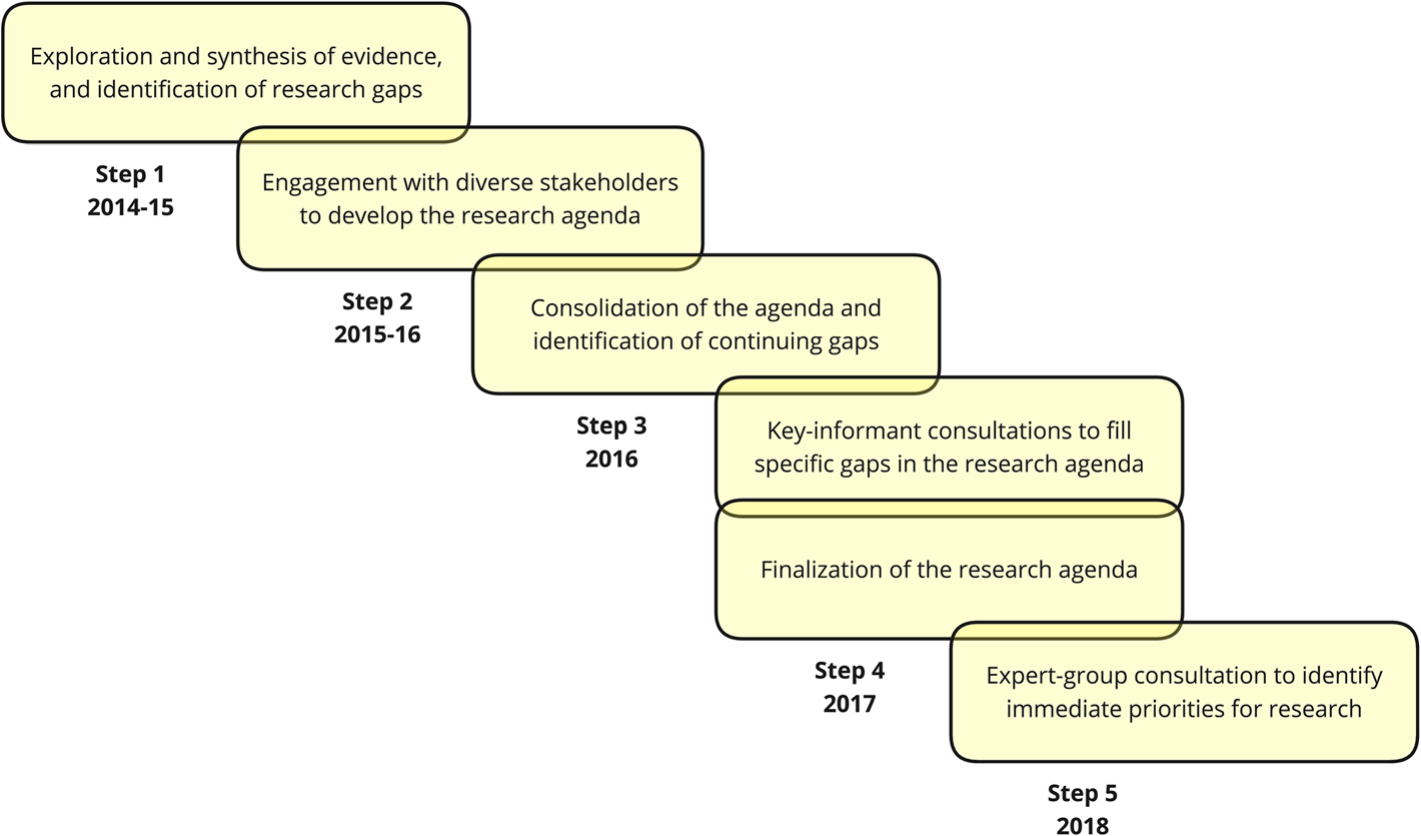
The five-step process adopted for the agenda- and priority-setting exercise between 2014 and 2018

We undertook the agenda- and priority-setting exercise over a 4-year period (2014–2018) using a five-step process (Fig. 1).

### Step 1: Exploration and synthesis of evidence

A detailed exercise was undertaken during 2014–2015 identifying public health research studies on health inequities in India, published during 2000–2014. The identification of studies was an iterative process, which included searches in major public health and social science databases and websites of organisations engaged with research on issues concerning the health of marginalised populations. We also consulted with researchers engaged in health equity research who pointed us to additional data sources. The search was limited to research available in English.

We developed a schema for the classification of health equity research across three dimensions, namely the nature of the research question, determinants of health inequities, and health conditions or outcomes studied (Table 1). This schema was arrived at after reviewing an initial set of studies identified for the synthesis. The schema was used to identify gaps in the evidence (Additional file 1). We found that most studies identified the existence of gaps in health outcomes associated with specific socioeconomic and demographic factors. Few studies went on to examine why and how these gaps came about. We also found that an overwhelming majority of the studies on health inequities were about maternal and child health. Of the five different axes or determinants of inequities examined, there was a dearth of studies on Dalit or Adivasi status, and evidence was especially scarce on health inequities experienced by vulnerable groups such as the elderly, persons living with physical and psycho-social disabilities, and persons of non-conforming sexual orientations and gender identities.

**Table 1:** Schema for organising the synthesis of research evidence and agenda

Dimension	Themes	Research agenda and questions
Dimension one	Type of research questions	I. Descriptive research that answers the ‘what’, ‘where’ and ‘when’ questions on the extent, nature and trends of health inequities II. Explanatory research that answers the ‘why’ and ‘how’ questions on the pathways through which health inequities are created, and the political/policy environment that facilitates the process III. Explanatory research that answers ‘how’ health systems create or facilitate inequities in accessibility, affordability, acceptability, and quality IV. Intervention research that answers the ‘what works in addressing health inequities, in which context, and why?’
Dimension two	Determinants of health inequities	I. Socioeconomic position II. Caste/tribal status III. Gender IV. Other socially constructed vulnerable groups, e.g. people living with HIV/AIDS, migrants, elderly, sexual minorities, sex workers, persons living with physical disabilities, persons living with mental health conditions V. Health systems
Dimension three	Health conditions/outcomes	I. Maternal health, wellbeing and nutrition II. Child health, wellbeing and nutrition III. Non-communicable diseases IV. Communicable diseases V. Violence and injuries VI. Overall mortality, morbidity, nutrition

The schema (Table 1) also formed the basis for an evidence synthesis exercise (published elsewhere), and for soliciting themes for further research from multiple stakeholders [6]. We then used the same schema to organise the research agenda that emerged from the present exercise.

### Step 2: Engagement with diverse stakeholders to develop the research agenda

This step lasted for approximately 1 year. Research gaps identified in Step 1 were presented to diverse stakeholders at two settings where research questions, areas and themes were gathered. The first was at a national seminar on health inequities held in August 2015. The seminar brought together approximately 60 researchers, civil society actors and policy-makers with track records of engaging with health equity issues. In a session dedicated for this purpose, participants studied the research gaps emerging from the evidence synthesis and generated a list of broad areas, themes and research questions individually. Contributions from all the participants were collected and compiled. The second was an online consultation through the health inequities web portal (http://www.healthinequity.com) to consult diverse constituencies. This web-portal of the Health Equity Research Initiative in India features regular updates on project relevant activities and newsletters. Once again, the research gaps were shared, and contributions to the research agenda were solicited by email. The web portal has a membership of approximately 450 researchers and activists from diverse sectors. 

### Step 3: Consolidation of the agenda and the identification of continuing gaps

The questions/themes generated through stakeholder engagement were consolidated and organised, once again, according to the schema described in Table 1. The consolidation led to the identification of continuing gaps in research questions related to health inequities. In particular, these were related to specific groups about whom little evidence is available related to health inequities, specifically persons living with physical and psychosocial disabilities and mental health problems, the elderly, persons of non-conforming gender identities or sexual orientations (Lesbian, Gay, Bisexual, Transgender, Queer and Intersex communities), and migrant populations.

### Step 4: Key-informant consultations to fill specific gaps in the research agenda; finalisation of the research agenda

In 2017, for each of the four areas for which research areas had not been identified through the earlier steps, specific persons with expertise in the identified areas were contacted by email or phone, briefed about the exercise and requested to contribute to the research agenda. We contacted at least 5–6 persons for each of the four areas, and research questions/themes were elicited in consultation with them.

At the end of Step 4, a list of 231 research questions/themes was prepared. Next, the research themes and questions were consolidated. The project team clubbed together similar questions, eliminated repetitions, distilled questions and themes for clarity, and organised all the questions according to dimension one of the initial schema, namely types of research questions.

Approximately 200 persons participated in steps 2 to 4. Of these, participants in the national seminar (Step 2) had the opportunity to have a face-to-face presentation on the evidence gaps, discuss with each other and hand-in their ideas on what should go into the research agenda. This group of approximately 60 people consisted predominantly of senior public health experts and senior civil society actors working on health equity, with many combining research and activism, and 10 policy-makers from the central and state governments. Approximately 120 people who participated in the online consultations (Step 2) were predominantly young and intermediate-career public health and social science researchers. The key informant interviews (Step 3) included approximately 20 researchers and civil society actors from key under-represented constituencies.

### Step 5: Expert group consultation to identify immediate priorities for research in health equity

From the medium-term (10-year) research agenda that we had generated, we wanted to identify a subset of immediate priorities for research. The basis for prioritising was one or more of the following: (1) potential for action; (2) potential for filling a crucial evidence gap; and (3) potential for expanding conceptual understanding of health inequities and pathways as well as processes contributing to it.

This last step consisted of an expert group consultation held at the National Conference on Health Inequities in India, organised by Achutha Menon Centre for Health Science Studies on January 8–11, 2018, at Trivandrum, India. A closed group consultation with experts was held as part of the Conference, in which 20 experts reviewed the research agenda. The experts were from diverse backgrounds and often wore several hats simultaneously. For example, many were civil society actors cum researchers, or mainly researchers who also engaged in advocacy and activism. In terms of disciplines, there were health systems researchers, epidemiologists, social scientists and anthropologists. In terms of areas of focus, there were persons working on issues related to caste- and ethnicity-based discrimination, gender and rights of persons living with mental health problems, disabilities and of persons with non-conforming gender identities and sexual orientation. We chose to prioritise through consensus, first within the small groups and then within the larger group of all 20 experts.

The expert group broke-out into four subgroups of five each. Each group reflected on one of the four categories of research questions and came up with priority areas or questions for immediate engagement. The priority areas and questions were usually drawn from the larger research agenda but were sometimes an addition to it. Each group discussed and arrived at a list of priorities, and then presented their top priorities in the large group. A list of immediate (next 2–3 years) priorities were identified in each research category through discussion and consensus in the group. 

In the next section, we present the research agenda and immediate research priorities for each of the four categories of research questions.

## Results

### Research agenda and priorities identified

The medium-term research agenda and priority questions that emerged from the 4-year consultative process were organised into the following four groups, based on the nature of the research question (Table 1).

1. Descriptive research that answers the ‘what’, ‘where’ and ‘when’ questions on the extent and nature and time trends of health inequities
2. Explanatory research that answers the ‘why’ and ‘how’ questions on the pathways through which health inequities are created and the political or policy environment that facilitates the process
3. Explanatory research that answers ‘how’ health systems facilitate or mitigate inequities in accessibility, affordability, acceptability and quality of healthcare
4. Intervention research that answers the question ‘what works in addressing health inequities, in which context, and why?’

In this section, we present the medium-term (10-year) research agenda organised according to the above categories and, within each category, we also present areas identified as immediate priorities.

#### I. Descriptive research that answers the ‘what’, ‘where’ and ‘when’ questions on the extent and nature and time trends of health inequities

We have termed ‘descriptive’ studies that describe the existence of health inequities, note the nature of the gaps across locations and over time. Many descriptive studies identify economic position, caste, tribal status or sex as correlates of differentials in health outcomes. Some of them also track changes (or lack thereof) in health outcomes over time. Many social groups experiencing vulnerabilities and marginalisation have not been the subject of descriptive studies on health inequities.

Descriptive studies are important to establish that specific population groups experience health inequities, and to motivate further studies into the reasons underlying the observed inequities. In the Indian context, there are several vulnerable population groups about whom such information is not available. The research agenda identified 12 groups who have been least represented in the evidence on health inequities, about whom it is important to initiate descriptive studies on health inequities. Studies are needed which describe the health situation of these groups, locating it in the context of population averages or comparing it with the health outcomes of groups known to enjoy greater power and privileges. For each of these groups, studies are needed on health behaviours as well as health outcomes. Table 2 presents the research agenda for descriptive studies on the least studied population groups.

**Table 2:** A research agenda for descriptive studies on least studied population groups

*Population groups to be studied* i. Muslims and other religious minorities ii. Nomadic tribes iii. Urban homeless iv. Migrants v. ‘Left behind’ households of migrants vi. Adolescents vii. Elderly viii. Single (never married/widowed/separated) ix. Persons living with physical/psychosocial disabilities x. Lesbian, Gay, Bisexual, Transgender, Queer, Intersex communities xi. Sex workers xii. People living with HIV/AIDS xiii. Communities living in the north-eastern states of India	*Health outcomes* i. Overall health needs of specific populations ii. Specific health conditions about which there is limited information (e.g. cervical cancer); specific health conditions in specific population groups (e.g. tuberculosis in elderly or internal migrants) iii. Ignored health needs of specific populations, e.g. beyond sexual and reproductive health for adolescents, beyond HIV for people living with HIV/AIDS iv. Nutritional status i. Quality of life, perceived psychological and physical wellbeing
	*Health behaviours* i. Health literacy/awareness of healthy behaviours and symptoms of health problems ii. Care-seeking behaviour (from whom, after how many days, for which conditions) iii. Access and utilisation, unmet need for healthcare/treatment compliance/treatment completion and barriers to these iv. Experience with healthcare providers/in healthcare facilities

The research agenda for descriptive studies on health inequities has a second part, which pertains to groups whose experience of health inequities is well established. Apart from some small-scale studies, there are a large number of studies analysing data from National Family Health Surveys, from Sample Registration Surveys and National Sample Surveys. For such population groups, the need is to go beyond the analysis of the next round of national surveys and to look at more complex themes even within descriptive studies.

The research agenda for more complex descriptive studies include the following:

1. Looking at within-group health inequities in vulnerable groups (e.g. within the group of Dalit*s* or Adivasis, of women and men, of low-income groups)
2. Examining the consequences to health inequities of intersections of multiple vulnerabilities (e.g. elderly by class and gender, adolescents by rural/urban location and age, migrants by rural-to-urban or urban-to-urban migration)
3. Changes over time and differences across geographic locations of health inequities (e.g. changes over time in caste-based or gender-based health inequities)
4. Comparing relative position in the social gradient of different marginalised groups (e.g. Dalits compared to Adivasis compared to Muslims)

The set of immediate priorities for descriptive research studies called for a focus on persons living with disabilities, on the different communities in the north-east region of India, on within-group stratification among Dalit and Adivasi groups, and on health conditions beyond maternal and child health. A more detailed list is presented in Table 3.

**Table 3:** Immediate priorities identified for various types of research

Type of research	Immediate priorities identified
I. Descriptive research	∙ Persons living with physical or psychosocial disabilities; their health conditions beyond their disabilities, such as sexual and reproductive health; within-group variations by marital status, caste/tribal status/religion/location/combinations of these (e.g. the health of women living with disabilities in tribal communities) ∙ Health inequities across different communities in north-east India and their particular contexts ∙ Health inequities experienced within Dalit and Adivasi populations, e.g. Valmikis as compared to better-off Dalit groups, nomadic tribal groups ∙ Lesbian, Gay, Bisexual, Transgender, Queer, Intersex (LGBTQI) communities: desk review on policy and law, and overall health status across states of India ∙ Studies on inequities in health conditions and utilisation of healthcare services beyond maternal and child health ∙ Features of curricula/pedagogies address sexuality and gender in medical and allied health professions ∙ Structural determinants of access to healthcare by workers in the informal sector (by gender, caste, age, geographic location) ∙ The burden of mental health of people who experienced violence (interpersonal/social/communal/conflict-related) across gender and age
II. Explanatory research related to social mechanisms and processes	In the immediate future, the focus needs to be on building theoretical, conceptual and methodological tools to make such research possible ∙ A conceptualisation of processes of inclusion, exclusion, discrimination, stigmatisation, marginalisation to better understand how social position results in unequal access to social determinants of health and health services ∙ Interfaces and interactions of macro-meso-microlevel factors in understanding health inequities ∙ Processes through which certain groups of people are rendered invisible in data. Alternatively, determinants of collection or non-collection of data on specific groups and categories of people, on some conditions versus others ∙ The process of evolving methodologies that capture the dynamics of health inequities without assuming static, timeless categories (for example, by caste, gender or economic position)
III. Explanatory research related to health system	∙ The impact of the growing presence of corporate private sector on access, availability, quality and affordability of healthcare ∙ The impact of philanthro-capitalism on global and national health governance, its consequences, and its impact on the corporate private sector ∙ The challenges in aligning bottom-up planning, top-down financing, and choice of technology (strategy and design), to assess if the tension between the three remain the same for groups across the social gradient ∙ Reasons why districts with similar levels of social determinants differ in terms of health system performance, features of governance that make the difference ∙ Perception of health workers/providers on the scope of community participation across the levels of the health system
IV. Intervention research	∙ Documentation of successful pilots, projects, innovations that have broken the barriers to equity and worked with the marginalised populations to see how some of them can be upscaled and integrated into the health system ∙ Type of interventions that worked or did not work for healthcare providers/health system to become responsive to specific needs of vulnerable groups (e.g., LGBTQI, migrants, people with disabilities, sex workers) ∙ Interventions that work to increase accountability to and participation by vulnerable groups ∙ Implementation and impact of Maternal Death Review for different populations (e.g. increased maternal death reporting, increased action taken over deaths reported, influence on the identification of ‘high-risk’ groups) ∙ Best practices of convergence models that bring out better health and nutrition outcomes especially of vulnerable groups ∙ Interventions that result in increasing the visibility and voice of marginalised groups ∙ Interventions that attract and retain workers to serve in marginalised areas

#### II. Explanatory research that answers the ‘why’ and ‘how’ questions, on the pathways through which health inequities are created, and the political/policy environment that facilitates the process

For some population groups, including Dalits, Adivasis, low-income groups, women in specific settings, residents of rural areas, urban slums or poorly performing districts or states, existing research has focused on the nature and extent of the disadvantage they experience but has seldom gone deeper so as to understand the reasons for the disadvantage. Studies within these groups need to shift gear and move towards explanatory studies. Concerning each of these population groups, the research agenda for explanatory studies on social processes leading to health inequities calls for a focus on questions that explain how health inequities have come about or are sustained. The two broad strands of questions are (1) what are the social processes that translate a specific social location into disadvantages in terms of access to resources and power, and through these, to poor health? (e.g. social exclusion, discrimination, stigma which may be the pathway through which Adivasi households are deprived of access to health resources), and (2) what are the macro-level socioeconomic and political determinants creating conditions that widen or narrow social stratification, contributing to health inequities? (e.g. cuts in public spending on the social sector, informalisation of labour, corporate control over healthcare).

The outcome variables to be examined are similar to those listed for descriptive studies, namely relevant behaviours and health outcomes, including morbidity, mortality and well-being, and access to and utilisation of healthcare and the quality of care received. As in the case of descriptive research studies, so also with explanatory research studies, the research agenda calls for exploring multiple axes of vulnerabilities. Explanatory research explores whether the mechanisms and processes underlying inequities differ for groups experiencing multiple disadvantages and provides valuable insight. However, to study mechanisms and underlying processes, research also needs to focus on building theoretical, conceptual and methodological tools. The key research priorities identified are summarised in Table 3.

#### III. Explanatory research that answers ‘how’ health systems create, reinforce or mitigate inequities

The third category of research questions regards the role of the health system in facilitating or mitigating health inequities. The purpose of health systems is to ensure a basic level of healthcare for all. However, health systems are also social institutions, embedded in the fabric of the society of which they are a part [9]. As a consequence, unless explicit and conscious policy measures are adopted, health systems are most likely to reflect the hierarchies and power relations of the context within which they are located. Thus, while health systems have the potential to uphold values of equitable and universal access, and respect the human rights of all its users, they could also reinforce and perpetuate health inequities and be blind to discrimination against vulnerable populations. For all these reasons, health systems are a crucial domain of inquiry when researching the mechanisms underlying health inequities.

Explanatory studies are needed on the role of health systems in caste, gender and socioeconomic status-based inequities in health in the poor health of various vulnerable population groups, similar to those identified for research questions in category two above. With respect to each of these population groups, the research agenda for explanatory studies on health inequities calls for a focus on questions that explain how health inequities have come about and are sustained. The two broad strands of questions are (1) how do the structure of the health system (e.g. public/private mix, distribution of services across levels of care, the extent of decentralisation, financing), the design of service delivery, the distribution of human and financial resources, and the processes of decision-making within the health system affect health inequities? (e.g. the requirement of residence permits may exclude migrant workers from accessing services, the lack of a woman doctor may discourage women from accessing gynaecological services of a sensitive nature); and (2) how do factors at the global and national levels influence the structure and functioning of the health system (e.g. government policies on the privatisation of healthcare, World Trade Organisation’s intervention to alter the pharmaceutical scenario, employment opportunities abroad for nurses).

The outcome variables of interest are accessibility, acceptability, affordability and quality of healthcare services and inequities, and their socioeconomic consequences in these across population groups. The key research priorities identified are summarised in Table 3.

#### IV. Intervention research that answers the question ‘what works in addressing health inequities, in which context, and why?’

Finally, moving from asking ‘what’, ‘why’ and ‘how’ questions for various population groups and existing health inequities, it is crucial for research to also support action in terms of developing or evaluating interventions that aim to address the health inequities identified. We classified the different interventions that deal with health inequities into the following categories: (1) interventions aimed at improving health outcomes (for instance, those aimed at reducing infant and maternal mortality); (2) interventions that target specific population groups (like children or elderly) or locations (like high priority districts); (3) interventions that attempt to improve awareness or influence health-related behaviours; and (4) interventions that aim to improve access to social determinants of health, thereby improving their health outcome (like access to better housing or nutritious food).

The research agenda on interventions research called for a focus on both descriptive and explanatory studies. There is a dearth of descriptive studies that explain in detail the health inequities addressed by the intervention, actors involved, strategies adopted, the theory of change of the intervention, challenges of implementation, and the outcomes in terms of success or failure in reducing the health inequities targeted.

The research agenda for explanatory studies on health equity interventions include studies that examine the reasons why some interventions succeed while others do not. Among the range of factors are the context, actors, strategies, implementation process and governance, not only at the local level but also at the macro level. The key research priorities identified for intervention research are summarised in Table 3.

The 4-year-long exercise described herein had many limitations. The research gaps identified were based on a synthesis exercise limited to studies published in the English language, which may have resulted in the exclusion of salient evidence published in other Indian languages. Further, the consultative process with stakeholders in a national seminar and through online calls for participation did not yield the expected results. Despite receiving more than 150 responses, some of the population groups on whom there was a major evidence gap were poorly represented in the research agenda, and we had to introduce an additional step of consultation with key informants to address this. Further, the short list of immediate priorities includes not only what emerged through the broad-based consultations but also specific concerns of a small group of experts. Finally, our consultations included civil society actors representing the interests and concerns of many vulnerable groups, and not the groups themselves, which would have called for resources that were not within the scope of our project.

Despite these limitations, the process adopted was consultative and inclusive, drew stakeholders from many disciplines including early and mid-career researchers and senior experts. The medium-term research agenda and immediate priorities for health equity research in India include a comprehensive range of research questions, ranging from the descriptive to the analytical, and encompassing intervention research. While much of the research agenda consists of empirical research questions, there was also an emphasis on theory-building based on findings on the ground. This was based on the finding from Step 1 of this exercise that public health research in health inequities seldom based itself on theoretical knowledge either from within the discipline or from other disciplines.

## Conclusion

The strength of this research agenda is that it was developed through a broad-based consultation with stakeholders representing diverse disciplines, sectors and constituencies. It aims to motivate and inspire public health researchers to ask the right questions, answers to which will help unravel the mechanisms and pathways contributing to health inequities, and to identify interventions that will contribute to redressing inequities. We also hope that those who sponsor and fund public health research will draw on this research agenda to identify key funding priorities on health inequities research, to generate evidence that will help India move closer to the SDG goal of leaving no one behind.

## Additional file

Additional file 1: Research gaps identified through the mapping and synthesis exercise. (PDF 33 kb)
