# Supplementary material for: A health equity research agenda for India: results of a consultative exercise
Source: Health Res Policy Syst. 2018 Oct 9;16(Suppl 1):94. doi: 10.1186/s12961-018-0367-0 (PMC6178245; doi:10.1186/s12961-018-0367-0)
Supplement: Supplementary file 1 — Research gaps identified through the mapping and synthesis exercise. (PDF 33 kb) [file 12961_2018_367_MOESM1_ESM.pdf]

Additional file 1

Research gaps identified through the mapping and synthesis exercise

The following research gaps in health equity research were identified through the mapping and syntheses exercises carried out:

1. Axes and major drivers of health inequities

- Class/socio-economic status: The vast majority of studies on health inequities have examined variations by consumption expenditure class or by standard of living index or wealth index. However, they have been focused mainly on maternal health and health of children under five years of age. Studies on inequities by socio-economic status in incidence/prevalence of communicable and non-communicable diseases, mental health or injuries are very few.
- Caste/ethnicity: There is a major gap in studies examining caste and tribe based inequities in health outcomes, health seeking behaviour and access to and affordability of health care services. The small number of studies is focused on maternal health and health of children under five.
- Gender: There are almost no studies examining gender-based differentials between women and men and boys and girls above age 5 in health status and in health seeking behaviour for health conditions common to both.
- “Socially constructed” vulnerabilities: A generally neglected area is inequities in health, experienced by specific population groups subject to discrimination and stigma in different contexts. This includes for example persons living with disabilities (physical and mental); persons living with specific stigmatised health conditions; migrant workers. The exception is Persons living with HIV/AIDS, a relatively better studied group.
- Health systems: Almost no studies examining how health systems as social institutions contribute to health inequities

- A big gap exists in terms of studies simultaneously examining multiple axes of social inequalities (e.g. urban and female and poor)

2. Inequities in health conditions

- Inequities in all reproductive health conditions besides pregnancy and delivery have remained little studied
- Few studies exist on inequities in communicable and non-communicable diseases, mental health, injuries and inequities in disabilities

3. Inequities across specific population groups - Missing from most studies are:

- Girls above 5 years of age and women outside the reproductive age group (above 45 years of age)
- Boys above 5 years of age and men of all ages
- Urban populations (as compared to rural)
- Occupational groups
- North-eastern states of India and Jammu and Kashmir, and relatively "developed" states such as Kerala, Tamil Nadu and Goa (as compared to other states)

4. Gaps in types of studies

- Studies based on primary data are few
- Studies use qualitative methods or mixed methods are few
- Studies that examine processes through which health disparities are created; or processes through which social inequalities translate into health inequities, are almost non-existent
- Another major gap is in terms of studies on interventions or policies that aim to close the gap in health status or access to health care services
